# Advanced electron crystallography through model-based imaging

**DOI:** 10.1107/S2052252515019727

**Published:** 2016-01-01

**Authors:** Sandra Van Aert, Annick De Backer, Gerardo T. Martinez, Arnold J. den Dekker, Dirk Van Dyck, Sara Bals, Gustaaf Van Tendeloo

**Affiliations:** a Electron Microscopy for Materials Research (EMAT), University of Antwerp, Groenenborgerlaan 171, B-2020 Antwerp, Belgium; b iMinds-Vision Lab, University of Antwerp, Universiteitsplein 1, B-2610 Wilrijk, Belgium; c Delft Center for Systems and Control (DCSC), Delft University of Technology, Mekelweg 2, 2628 CD Delft, The Netherlands

**Keywords:** transmission electron microscopy, quantitative analysis, statistical parameter estimation, experimental design, structure refinement

## Abstract

The increasing need for precise determination of the atomic arrangement of non-periodic structures in materials design and the control of nanostructures explains the growing interest in quantitative transmission electron microscopy. The aim is to extract precise and accurate numbers for unknown structure parameters including atomic positions, chemical concentrations and atomic numbers. For this purpose, statistical parameter estimation theory has been shown to provide reliable results. In this theory, observations are considered purely as data planes, from which structure parameters have to be determined using a parametric model describing the images. As such, the positions of atom columns can be measured with a precision of the order of a few picometres, even though the resolution of the electron microscope is still one or two orders of magnitude larger. Moreover, small differences in average atomic number, which cannot be distinguished visually, can be quantified using high-angle annular dark-field scanning transmission electron microscopy images. In addition, this theory allows one to measure compositional changes at interfaces, to count atoms with single-atom sensitivity, and to reconstruct atomic structures in three dimensions. This feature article brings the reader up to date, summarizing the underlying theory and highlighting some of the recent applications of quantitative model-based transmisson electron microscopy.

## Abstract 2

An overview of statistical parameter estimation methods is presented and applied to analyse transmission electron microscopy images in a quantitative manner.

## 1. Introduction   

New developments in the field of nanoscience and nanotechnology drive the need for advanced quantitative materials characterization techniques that can be applied to complex nanostructures. The physical properties of nanostructures, including electrical, mechanical and chemical properties, are obviously controlled by the composition and chemical bonding, but also by the exact positions of the atoms. Because of the presence of defects, interfaces and surfaces, the locations of the atoms of nanostructures deviate from their equilibrium bulk positions. This results in strain playing a crucial role in the observed properties. For example, strain induced by the lattice mismatch between a substrate and a superconducting layer grown on top can change the interatomic distances by picometres and can in this manner turn an insulator into a conductor (Locquet *et al.*, 1998 ▸). In order to unscramble the structure–properties relation, experimental characterization methods are thus required that can locally determine the unknown structure parameters with sufficient precision (Spence, 1999 ▸; Muller & Mills, 1999 ▸; Springborg, 2000 ▸; Olson, 2000 ▸). A precision of the order of 0.01–0.1 Å is needed for the atomic positions (Muller, 1999 ▸; Kisielowski, Principe *et al.*, 2001 ▸). If we can determine the type and position of the atoms with sufficient precision, the atomic structure can be linked to the physical properties. A common approach to understanding materials properties is the use of theoretical *ab initio* calculations that allow one to obtain equilibrium atomic positions for a given composition. Once this equilibrium structure has been obtained the physical properties can be computed and predictions of how the material would behave under different environmental conditions, even beyond the capability of any laboratory, can be performed. In this manner, materials science is gradually evolving towards materials design, *i.e.* from describing and understanding towards predicting materials with interesting properties (Wada, 1996 ▸; Olson, 1997 ▸, 2000 ▸; Reed & Tour, 2000 ▸; Browning *et al.*, 2001 ▸).

**Figure 1:**
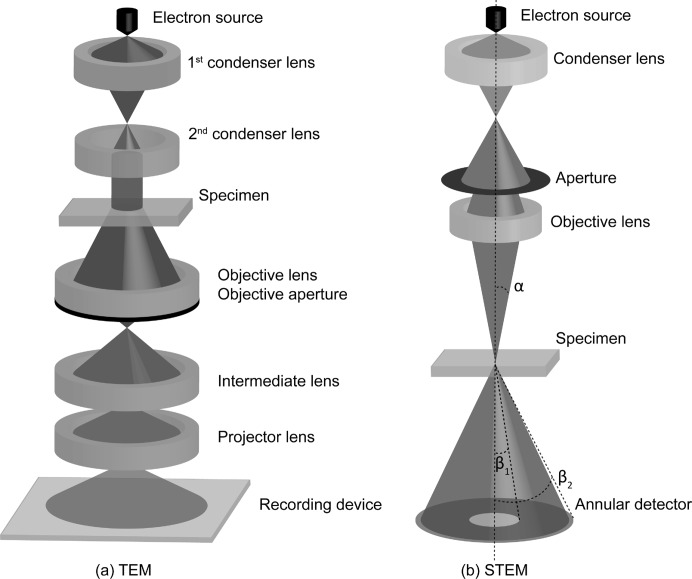
General schematics of (*a*) a TEM and (*b*) a STEM instrument. (*a*) A plane wave illuminates the object, after which an image is formed using a set of electromagnetic lenses. (b) An electron beam with convergence angle α is scattered by the specimen and collected by an annular detector with inner and outer angles β_1_ and β_2_, respectively.

Transmission electron microscopy (TEM) is an excellent technique to study nanostructures. Compared with X-rays, electrons interact very strongly with small volumes of matter, providing local information on the material under study (Zanchet *et al.*, 2001 ▸; Spence, 1999 ▸; Henderson, 1995 ▸). In this manner, TEM can be used to observe deviations from perfect crystallinity, which is of great importance when studying nanostructures. Fig. 1 ▸ gives a schematic overview of two commonly used imaging modes, namely conventional TEM and scanning transmission electron microscopy (STEM). In TEM, the object is illuminated by a parallel incident electron beam which is formed by a set of condenser lenses. An image of the object under study is produced which is then magnified by the remaining imaging lenses and projected onto the viewing device, which is usually a charge-coupled device (CCD) counting the electrons reaching the camera. In the STEM imaging mode, an electron beam is focused to a fine probe that is scanned across the sample in a two-dimensional raster (Crewe, 1968 ▸; Crewe *et al.*, 1970 ▸; Nellist & Pennycook, 2000 ▸). For each probe position, the electrons scattered towards the detector are integrated and displayed as a function of probe position. Different detector geometries are available nowadays. Depending on the collection range of the detector, the dependence of the contrast on the atomic number *Z* will be different, as will the pixel signal-to-noise ratio (Cowley *et al.*, 1995 ▸; Hovden & Muller, 2012 ▸). Both imaging modes, TEM and STEM, result in two-dimensional projected images of three-dimensional objects.

Over the past few years, remarkable high-technology developments in lens design have greatly improved image resolution. Currently, a resolution of the order of 50 pm can be achieved (Haider *et al.*, 1998 ▸; Urban, 2008 ▸; Jia *et al.*, 2008 ▸; Jia, Mi, Faley *et al.*, 2009 ▸; Erni *et al.*, 2009 ▸). For most atomic types, this exceeds the point where the electrostatic potential of the atoms is the limiting factor. Furthermore, new data collection geometries are emerging that allow one to optimize the experimental settings (Shibata *et al.*, 2010 ▸; Hovden & Muller, 2012 ▸; Gonnissen *et al.*, 2014 ▸; De Backer, De wael *et al.*, 2015 ▸; Yang *et al.*, 2015 ▸). In addition, detectors behave more and more as ideal quantum detectors. In this manner, the microscope itself becomes less restricting and the quality of the experimental images is set mainly by the unavoidable presence of electron counting noise. However, these images need to be interpreted quantitatively when aiming for precise structural information. Therefore, the focus in (S)TEM research is now gradually moving from obtaining better resolution to improving the precision with which unknown structure parameters, such as the atomic positions and atomic types, can be extracted from (S)TEM images. To reach this goal, the use of statistical parameter estimation theory is of great help (den Dekker *et al.*, 2005 ▸; Van Aert *et al.*, 2005 ▸, 2009 ▸, 2011 ▸, 2012 ▸, 2013 ▸; Bals *et al.*, 2006 ▸, 2011 ▸, 2012 ▸; De Backer *et al.*, 2011 ▸; De Backer, Martinez *et al.*, 2015 ▸; Martinez, Rosenauer *et al.*, 2014 ▸; Kundu *et al.*, 2014 ▸).

The purpose of this feature article is twofold. First, a concise overview of the methods that can be applied for the solution of a general type of parameter estimation problem often met in materials characterization or applied science and engineering will be presented. Second, applications of these methods in the field of TEM will be discussed. In these applications, the goal is to determine unknown structure parameters, including atomic positions, chemical concentrations and atomic numbers, as precisely as possible from experimentally recorded images. By means of examples, it will be shown that statistical parameter estimation theory allows one to measure two-dimensional atomic column positions with subpicometre precision, to measure compositional changes at interfaces, to count atoms with single-atom sensitivity and to reconstruct three-dimensional atomic structures.

## 2. Model-based parameter estimation   

In general, the aim of statistical parameter estimation theory is to determine, or more correctly to estimate, unknown physical quantities or parameters on the basis of observations that are acquired experimentally (van den Bos, 2007 ▸). In electron microscopy, these observations are, for example, the image pixel values recorded using a CCD camera. In this field as well as in many other scientific disciplines, such observations are not themselves the quantities to be measured but are related to the quantities of interest. Often this relation is a known mathematical function derived from physical laws and the quantities to be determined are parameters of this function. For example, if electron microscopy observations are made of a specific object, this parametric model should include all the ingredients needed to perform a computer simulation of the images, *i.e.* the electron–object interaction, the transfer of the electrons through the microscope and image detection. If models based on first principles cannot be derived, or are too complex for their intended use, simplified empirical models may be used. Some of the model’s parameters are the atomic positions and atomic types. The parameter estimation problem then becomes a case of computing the atomic positions and atomic types from electron microscopy images. Statistical parameter estimation theory provides an elegant solution for such problems. Indeed, based on the availability of a parametric model, the unknown structure parameters can be estimated by fitting the model to the experimental images in a refinement procedure, usually called an estimation procedure or *estimator*. In general, different estimation procedures can be used to estimate the unknown parameters of this model, such as least-squares (LS), least absolute deviations or maximum likelihood (ML) estimators (den Dekker *et al.*, 2005 ▸, 2013 ▸; van den Bos, 2007 ▸). In practice, the ML estimator is often used since it is known to be the most precise. This estimator will briefly be reviewed in §2.1, whereas the limits to precision will be discussed in §2.2. For a detailed overview of statistical parameter estimation theory, the reader is referred to den Dekker *et al.* (2005 ▸, 2013 ▸), van den Bos (2007 ▸), van den Bos & den Dekker (2001 ▸), Seber & Wild (1989 ▸) and Cramér (1946 ▸). The mathematics of statistical parameter estimation theory is given in the highlighted literature and instead this paper concentrates on the general concepts.

### 2.1. Maximum likelihood estimation   

As one will readily admit, any experiment involves at least some ‘noise’. This means that, if a particular experiment is repeated under the same conditions, the resulting observations will vary from experiment to experiment and so will the parameter estimates, which are computed on the basis of these observations. If the amount of variation in these estimates is small, we say that the corresponding estimator has a high precision. Accuracy refers to the absence of any systematic deviation of the parameter estimates from the true parameters, and thus to the absence of bias. Because of the unavoidable presence of noise in an experiment, the description of observations by means of only a deterministic parametric mathematical function, as discussed above, is insufficient since it does not account for the random fluctuations caused by the noise in the experiment. An efficient way of describing this behaviour is by means of statistics. This implies that the observations are modelled as so-called stochastic variables. By definition, a stochastic variable is characterized by its probability (density) function [P(D)F], while a set of stochastic variables has a joint P(D)F. Well known P(D)Fs are the Poisson distribution, which applies in the case of electron counting results, and the normal distribution, which can be used when disturbances other than pure counting statistics also contribute or when the electron dose is sufficiently large (Papoulis, 1965 ▸; Herrmann, 1997 ▸; Koster *et al.*, 1987 ▸). The joint P(D)F defines the expectations, *i.e.* the mean value of each observation and the fluctuations about these mean values. In a sense, the expectations would be the image pixel values recorded in the absence of noise and they can be modelled using a parametric model. The availability of this parametric model makes it possible to parameterize the P(D)F of the observations, which is of vital importance when quantifying the attainable precision with which unknown structure parameters can be estimated and when determining parameters using the ML estimator.

Different estimators that can be used to estimate the same unknown parameters will in general result in a different precision. However, the variance of unbiased estimators will never be lower than the so-called Cramér–Rao lower bound (CRLB), which is a theoretical lower bound on the variance (van den Bos, 1982 ▸; van den Bos & den Dekker, 2001 ▸; den Dekker *et al.*, 2013 ▸, 2005 ▸). The ML estimator achieves this theoretical lower bound asymptotically, *i.e.* for an increasing number of observations, and is therefore of practical importance. From the joint P(D)F of the observations, the ML estimator can be derived relatively easily. By substituting the experimental observations in the expression of the P(D)F and considering the structure parameters as random variables, the so-called likelihood function is obtained. Maximum likelihood estimated parameters are then obtained by maximizing this likelihood function. Note that the ML estimator equals the LS estimator for independent normally distributed observations, which is often a realistic assumption as discussed before (den Dekker *et al.*, 2005 ▸).

The search for the maximum of the likelihood function is an iterative numerical procedure and it requires a starting structure for the parameters which is sufficiently close to the real structure to avoid the risk of ending up in a local maximum (Möbus *et al.*, 1998 ▸). In electron microscopy applications, the dimension of the parameter space is usually very high. Consequently, it is quite possible that the optimization procedure ends up at a local maximum instead of at the global maximum of the likelihood function, so that the wrong structure parameters are suggested, which introduces bias. To solve this dimensionality problem, *i.e.* to find a pathway to the maximum in the parameter space, good starting values for the parameters are required (Van Dyck *et al.*, 2003 ▸). In other words, the structure has to be resolved. This corresponds to X-ray crystallography, where one first has to resolve the structure and afterwards refine it. Although it is not always trivial to resolve the structure, recent developments in aberration-corrected electron microscopy and the use of direct methods to invert the imaging process, such as focal-series reconstruction or electron holography, have resulted in great improvements (Van Dyck & Coene, 1987 ▸; Lichte, 1986 ▸; Van Dyck *et al.*, 1993 ▸; Kirkland *et al.*, 1995 ▸; Haigh *et al.*, 2009 ▸).

### 2.2. Attainable precision   

When estimating unknown structure parameters in a quantitative manner using statistical parameter estimation theory, we are not so much interested in the electron microscopy images as such, but rather in the (structural and chemical) information of the sample under study. Images are then considered as data planes, from which sample structure parameters, such as atomic positions, particle sizes and fibre diameters, have to be estimated as precisely as possible. Image quality and resolution are then required to resolve the structure but are no longer the ultimate goal.

Often, the question arises of how to measure atomic positions with picometre precision if the resolution of the instrument is ‘only’ 50 pm under optimal conditions. Resolution and precision are very different notions (van den Bos & den Dekker, 2001 ▸). In (S)TEM, resolution expresses the ability to distinguish visually between neighbouring atomic columns in an image. Classical resolution criteria, such as Lord Rayleigh’s, are derived from the assumption that the human visual system needs a minimum contrast to discriminate two points in its composite intensity distribution (Lord Rayleigh, 1902 ▸). Therefore, they are expressed in terms of the width of the point spread function of the (S)TEM imaging system (O’Keefe, 1992 ▸). However, if the physics behind the image formation process is known, images no longer need to be interpreted visually. Instead, atomic column positions can be estimated by fitting this known parametric model to an experimental image (den Dekker *et al.*, 2005 ▸; Van Aert *et al.*, 2005 ▸, 2006 ▸). In the absence of noise, this procedure would result in infinitely precise atomic column locations. However, since detected images are never noise-free, model fitting never results in a perfect reconstruction, thus limiting the statistical precision with which the atom locations can be estimated. For continuous parameters, such as the atomic column positions, the attainable precision can be adequately quantified from the joint PDF using the expression for the CRLB. Under certain assumptions, it can then be shown that the attainable precision, expressed in terms of the standard deviation with which the position of a projected atomic column can be estimated, is proportional to the instrumental resolution and inversely proportional to the square root of the number of detected electrons (Bettens *et al.*, 1999 ▸; Van Aert, den Dekker, Van Dyck & van den Bos, 2002 ▸; Van Aert, den Dekker, van den Bos & Van Dyck, 2002 ▸). This explains why the precision to estimate projected atomic column positions can be down to one or a few picometres, although the resolution of modern instruments is 50–100 pm. For instance, if one wants to obtain the position of an atom with a precision of the order of 1 pm, one will need an incident dose of electrons of the order of 1000 e Å^−2^. In order to push the precision further by a factor of 10, it is necessary to increase the dose by a factor of 100, which will require a very high brightness and/or a long exposure time.

For discrete parameters, such as atomic column types or numbers of atoms, the expression for the CRLB can no longer be used to compute the attainable precision. Recently, it has been shown that statistical detection theory provides an alternative solution to evaluate the performance to estimate discrete parameters (Kay, 2009 ▸; den Dekker *et al.*, 2013 ▸; Gonnissen *et al.*, 2014 ▸; De Backer, De wael *et al.*, 2015 ▸). For example, when considering the problem of identifying the atomic number (*Z*) from a STEM image of a single atom, the parameter to be estimated is a positive integer, in which case the CRLB is not defined. However, in the present problem of identifying *Z*, *a priori* knowledge concerning possible solutions for the atomic numbers is usually available. In such cases, the question reduces to distinguishing between a finite plausible set of values for the atomic numbers *Z* given the experimental STEM observations. Detection theory then provides the tools to decide between 2 or more hypotheses – where each hypothesis corresponds to the assumption of a specific *Z* value – and to predict the probability of assigning an incorrect hypothesis. This expression for the probability of error gives insight into the performance to make a correct decision and the sensitivity of this detection performance to the experimental settings (Gonnissen *et al.*, 2014 ▸; den Dekker *et al.*, 2013 ▸).

In the following sections, applications of statistical parameter estimation theory in the field of quantitative (S)TEM imaging are outlined.

## 3. Quantitative atomic column position measurements   

Aberration-corrected TEM, exit wave reconstruction methods or combinations of both are often used to measure shifts in atomic positions. Whereas aberration correction has an immediate impact on the resolution of the experimental images, the purpose of exit wave reconstruction is to retrieve the complex electron wavefunction which is formed at the exit plane of the sample under study. In practice, the exit wave is usually reconstructed from a series of images taken at different defocus values, from an electron holographic image or from a series of images recorded with different illuminating beam tilts (Van Dyck & Coene, 1987 ▸; Lichte, 1986 ▸; Van Dyck *et al.*, 1993 ▸; Kirkland *et al.*, 1995 ▸; Haigh *et al.*, 2009 ▸). Ideally, the exit wave is free from any imaging artifacts, thus enhancing the visual interpretability of the atomic structure. Because of its potential to visualize light atomic columns, such as oxygen or nitrogen, with atomic resolution, exit wave reconstruction has become a powerful tool in high-resolution TEM (Coene *et al.*, 1992 ▸; Kisielowski, Hetherington *et al.*, 2001 ▸). Although such reconstruction was often considered as a final result used to interpret the structure visually, its combination with quantitative methods nowadays demonstrates its potential to measure atomic column positions precisely (Jia & Thust, 1999 ▸; Ayache *et al.*, 2005 ▸; Bals *et al.*, 2006 ▸). As an example, the quantification of localized displacements at a {110} twin boundary in orthorhombic CaTiO_3_ will be discussed (Van Aert *et al.*, 2012 ▸).

**Figure 2:**
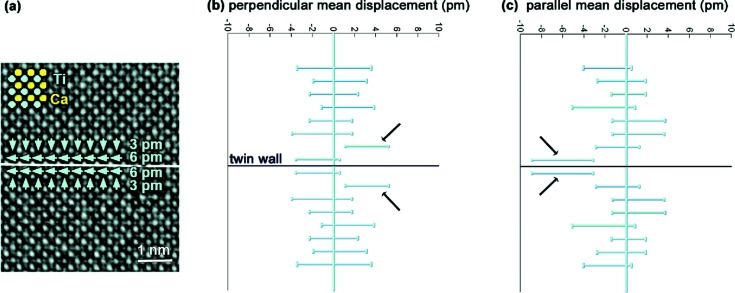
(*a*) Experimental phase image of a (110) twin boundary in orthorhombic CaTiO_3_. Mean displacements of the Ti atomic columns from the centre of the four neighbouring Ca atomic columns are indicated by green arrows. (*b*) and (*c*) Displacements of Ti atomic columns perpendicular and parallel to the twin wall, averaged along and in mirror operation with respect to the twin wall, together with their 90% confidence intervals. Reproduced from Van Aert *et al.* (2012 ▸), copyright 2011 John Wiley and Sons.

Numerical calculations have shown that domain boundaries in CaTiO_3_ are mainly ferrielectric with maximum dipole moments at the wall. Twin boundaries of the {110} type in orthorhombic CaTiO_3_ (space group *Pnma*) have been imaged along [001] using aberration-corrected TEM in combination with exit wave reconstruction. The phase of the reconstructed exit wave is shown in Fig. 2 ▸(*a*) with a resolution of 0.8 Å. This phase is directly proportional to the projected electrostatic potential of the structure. In order to obtain quantitative numbers for the atomic column positions, statistical parameter estimation is needed (den Dekker *et al.*, 2005 ▸; Van Aert *et al.*, 2005 ▸; van den Bos & den Dekker, 2001 ▸). This allows position measurements of all atomic columns with a precision of a few picometres without being restricted by the information limit of the microscope. Therefore, the phase of the reconstructed exit wave is considered as a data plane from which the atomic column positions are estimated in a statistical way. As discussed in §2, the key to successful application of statistical parameter estimation theory is the availability of a parametric model describing the expectations of the pixel values in the reconstructed phase. Nowadays, the physics behind the electron–object interaction is sufficiently well understood to have such a parameterized expression. The parameters of this function, including the atomic column positions, can then be determined using the LS estimator. From the thus estimated atomic column positions, it has been found that shifts in the Ti atomic positions in the vicinity of the twin wall are present, whereas possible shifts in the Ca atomic positions are too small to be identified (Van Aert *et al.*, 2012 ▸). Therefore, this analysis is focused on the off-centring of the Ti atomic positions with respect to the centre of the neighbouring four Ca atomic positions.

First, we average all displacements in planes parallel to the twin wall. Next, we average the results in the planes above with the corresponding planes below the twin wall. This second operation identifies the overall symmetry of the sample, with the twin wall representing a mirror plane. The resulting displacements along and perpendicular to the twin wall are shown in Figs. 2 ▸(*b*) and 2 ▸(*c*), respectively, together with their 90% confidence intervals. In the direction perpendicular to the wall, systematic deviations for Ti of 3.1 pm in the second closest layers pointing towards the twin wall are found. A larger displacement is measured in the direction parallel to the wall in the layers adjacent to the twin wall. The average displacement in these layers is 6.1 pm. In layers further away from the twin wall, no systematic deviations are observed. These experimental results confirm the theoretical predictions (Goncalves-Ferreira *et al.*, 2008 ▸). The thickness of the domain wall is about two octahedra. The displacement pattern can be seen as a combination of ferroelectric and antiferroelectric/ferrielectric components. The ferroelectric component is the smaller one and has an effect both parallel and perpendicular to the wall.

We can also calculate the magnitude of the spontaneous polarization of the wall. In the model calculations it was found that the wall polarization is between 0.004 and 0.02 C m^−2^. Using the experimental value for the displacement of 6 pm, we expect a polarization of the order of 0.04–0.2 C m^−2^. This value is comparable with the bulk spontaneous polarization of BaTiO_3_ (0.24 C m^−2^). The importance of this study is that localized effects can be quantified in combination with statistical parameter estimation theory, proving that ferro­electricity is indeed confined to twin boundaries in a paraelectric matrix.

Another efficient technique to measure shifts in atomic positions is so-called negative spherical aberration imaging, in which the spherical aberration constant *C*_s_ is tuned to negative values by employing an aberration corrector (Jia *et al.*, 2003 ▸, 2010 ▸). Compared with traditional positive *C*_s_ imaging, this imaging mode yields a negative phase contrast of the atomic structure, with atomic columns appearing bright against a darker background. For thin objects, this leads to a substantially higher contrast than for the dark-atom images formed under positive *C*_s_ imaging. This enhanced contrast has the effect of improving the measurement precision of the atomic positions and explains the use of this technique to measure atomic shifts of the order of a few picometres. Examples are measurements of the width of ferroelectric domain walls in PbZr_0.2_Ti_0.8_O_3_ (Jia *et al.*, 2008 ▸), measurements of the coupling of elastic strain fields to polarization in PbZr_0.2_Ti_0.8_O_3_/SrTiO_3_ epitaxial systems (Jia, Mi, Urban *et al.*, 2009 ▸) and measurements of oxygen-octahedron tilt and polarization in LaAlO_3_/SrTiO_3_ interfaces (Jia, Mi, Faley *et al.*, 2009 ▸).

## 4. Quantitative composition analysis   

Depending on the shape and size of the STEM detector, different signals can be recorded (Cowley *et al.*, 1995 ▸; Shibata *et al.*, 2010 ▸; Yang *et al.*, 2015 ▸). A key imaging mode is high-angle annular dark-field (HAADF) STEM, in which an annular detector is used with a collection range outside the illumination cone. The high-angle scattering thus detected is dominated by Rutherford and thermal diffuse scattering. Therefore, the HAADF signal scales approximately with the square of the atomic number *Z*, hence the name *Z*-contrast. One of the advantages is thus the possibility of distinguishing visually between chemically different atomic column types. Because of the incoherent imaging nature, the resolution observed in an HAADF STEM image is, to a large extent, determined by the intensity distribution of the illuminating probe. The use of aberration-corrected probe-forming optics currently gives a probe size of the order of 50 pm (Erni *et al.*, 2009 ▸). The combination of a high spatial resolution with a high chemical sensitivity makes HAADF STEM a very attractive tool for structure characterization at the atomic level.

Even though HAADF STEM images are to a certain extent interpretable directly, this imaging technique also benefits greatly from quantitative analysis using statistical parameter estimation theory (Van Aert *et al.*, 2009 ▸). This is particularly the case when the difference in atomic number of distinct atomic column types is small, or if the signal-to-noise ratio becomes poor. A performance measure which is sensitive to the chemical composition is the so-called scattering cross-section (Retsky, 1974 ▸; Isaacson *et al.*, 1979 ▸; Singhal *et al.*, 1997 ▸; Van Aert *et al.*, 2009 ▸; MacArthur *et al.*, 2013 ▸). Using statistical parameter estimation theory, the total intensity of scattered electrons can be quantified atomic column by atomic column using an empirical parameterized incoherent imaging model. The advantage of using scattering cross-sections over other metrics, such as peak intensities, is their robustness to magnification, defocus, source size, astigmatism and other aberrations, and small sample mis-tilt (MacArthur *et al.*, 2013 ▸, 2015 ▸; Martinez, De Backer *et al.*, 2014 ▸). The estimated scattering cross-sections allow us to differentiate between atomic columns with different compositions. As such, differences in average atomic number of only 3 can clearly be distinguished in an experimental image, which is impossible by means of visual interpretation alone. This is an important advantage when studying interfaces, as illustrated in the following example.

**Figure 3:**
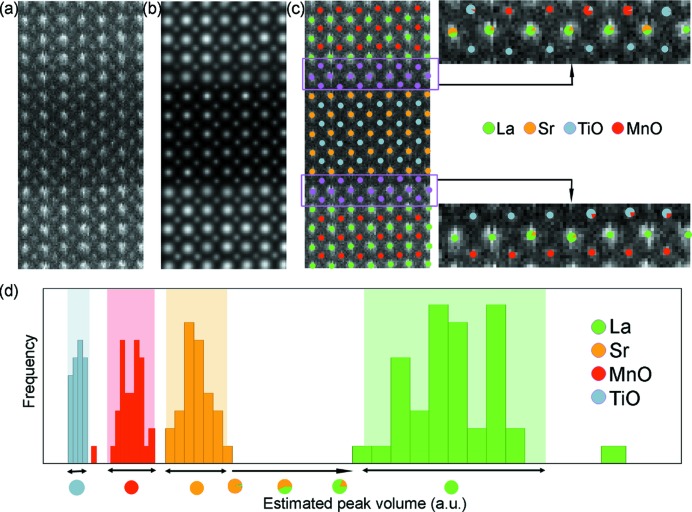
(*a*) Area from an experimental HAADF STEM image of an La_0.7_Sr_0.3_MnO_3_–SrTiO_3_ multilayer structure. (*b*) The refined parameterized model. (*c*) Overlay indicating the estimated positions of the columns, together with their atomic column types. (*d*) Histograms of the estimated scattering cross-sections of the known columns. Reproduced from Van Aert *et al.* (2009 ▸), copyright 2009 Elsevier.

Fig. 3 ▸(*a*) shows an enlarged area from an experimental HAADF STEM image of an La_0.7_Sr_0.3_MnO_3_–SrTiO_3_ multilayer structure using an FEI Titan^3^ 50-80 operated at 300 kV. Even though the probe has been corrected for spherical aberration, no visual conclusions could be drawn concerning the sequence of the atomic planes at the interfaces. The refined parameterized model is shown in Fig. 3 ▸(*b*), showing a close match with the experimental data. Fig. 3 ▸(*c*) shows the experimental observations, together with an overlay indicating the estimated positions of the columns and their atomic column types. The composition of the columns away from the interfaces is assumed to be in agreement with the composition in the bulk compounds, whereas the composition of the columns in the planes close to the interface (shown in purple) is unknown. Histograms of the estimated scattering cross-sections of the known columns are presented in Fig. 3 ▸(*d*) and show the random nature of the result. The coloured vertical bands correspond to 90% tolerance intervals. It is important to note that these tolerance intervals do not overlap, meaning that columns, for which the difference in average atomic number is only 3 (TiO and MnO) in this example, can clearly be distinguished. Based on this histogram, the composition of the unknown columns can be identified, as shown on the right-hand side of Fig. 3 ▸(*c*). Single-coloured dots are used to indicate columns whose estimated scattering cross-section falls inside a tolerance interval, whereas pie charts, indicating the presence of intermixing or diffusion, are used otherwise.

**Figure 4:**
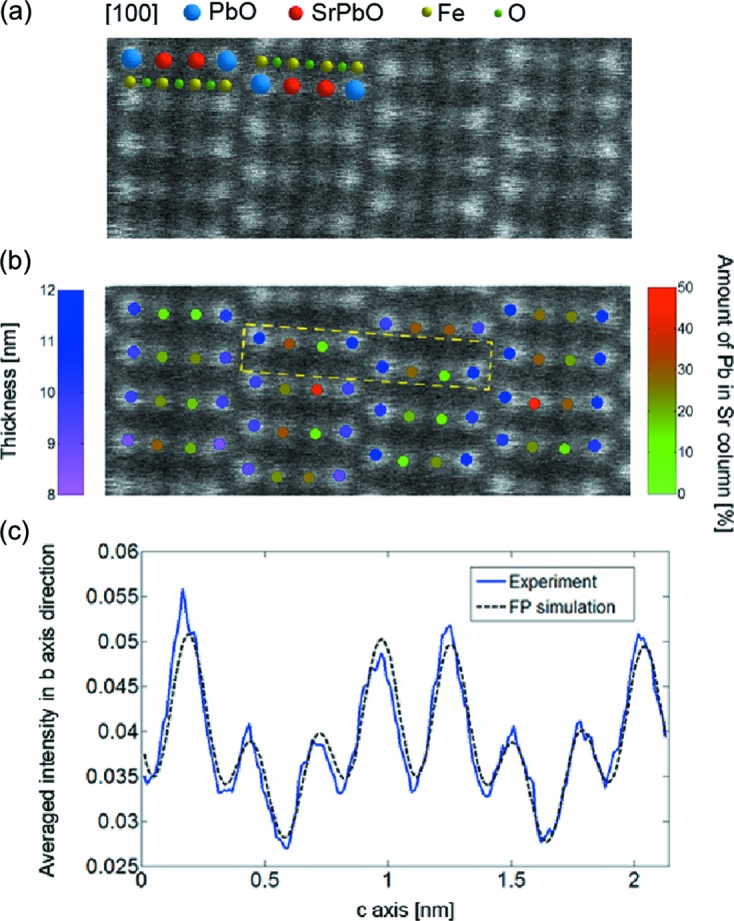
(*a*) Area from an experimental HAADF STEM image of a Pb_1.2_Sr_0.8_Fe_2_O_5_ compound, where the intensities are normalized with respect to the incoming electron beam. (*b*) Quantification results showing the estimated thickness values at the PbO site and the estimated composition for the SrPbO atomic columns. (*c*) Comparison of the average experimental intensity profile along the vertical direction of the unit cell indicated in part (*b*), together with a frozen lattice simulation assuming the thickness and composition values shown in part (*b*). Reproduced from Martinez, Rosenauer *et al.* (2014 ▸), copyright 2009 Elsevier.

The previous example shows how statistical parameter estimation theory can help to quantify the chemical composition in a relative manner. When aiming for an absolute quantification, intensity measurements relative to the intensity of the incoming electron beam are required (LeBeau *et al.*, 2008 ▸; Rosenauer *et al.*, 2009 ▸). In this manner, experimental scattering cross-sections can be compared directly with simulated scattering cross-sections (Rosenauer *et al.*, 2009 ▸; LeBeau *et al.*, 2010 ▸; Martinez, Rosenauer *et al.*, 2014 ▸). Reference cross-section values are then simulated by carefully matching experimental imaging conditions for a range of sample conditions, including thickness and composition. To illustrate this, Fig. 4 ▸(*a*) shows part of a normalized image of a Pb_1.2_Sr_0.8_Fe_2_O_5_ compound where the intensities are normalized with respect to the incoming electron beam (Martinez, Rosenauer *et al.*, 2014 ▸). By comparing experimental scattering cross-sections for each atomic column with simulated values, the thickness values for the PbO columns and the composition of the SrPbO columns have been determined, as shown in Fig. 4 ▸(*b*). Fig. 4 ▸(*c*) compares the average experimental intensity profile along the vertical direction of the unit cell indicated in Fig. 4 ▸(*b*) with a frozen lattice simulation, where the estimated thickness and composition values have been used as input. The overall match between the simulated and experimental image intensities further confirms the results that have been obtained when using the scattering cross-sections approach. However, it should be noted that small deviations between simulated and experimental image intensities can not be avoided because of, for example, remaining uncertainties in the microscope settings such as defocus, source size or astigmatism.

## 5. Nanoparticle atom counting   

**Figure 5:**
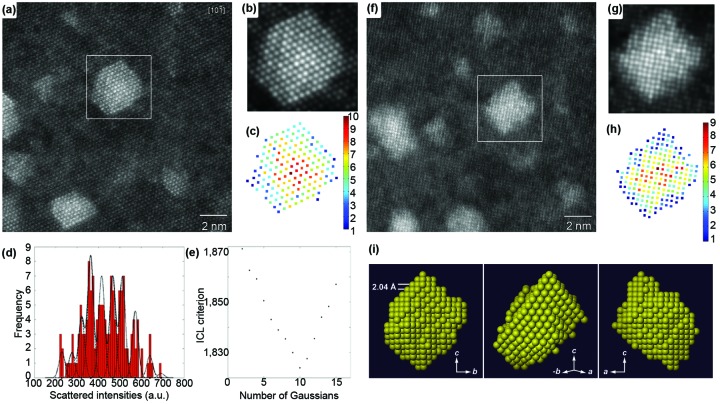
(*a*) Experimental HAADF STEM image of nanosized Ag clusters embedded in an Al matrix in 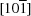 zone-axis orientation. (*b*) Refined parameterized model of the boxed region of part (*a*). (*c*) The number of Ag atoms per column. (*d*) Histogram of scattering cross-sections of the Ag columns. (*e*) Integrated classification likelihood (ICL) criterion evaluated as a function of the number of Gaussians in a mixture model. (*f*) Experimental HAADF STEM image in [100] zone-axis orientation. (*g*) Refined model of the boxed region of part (*f*). (*h*) The number of Ag atoms per column. (*i*) A computed three-dimensional reconstruction of the Ag nanocluster, viewed along three different directions. Reproduced from Van Aert *et al.* (2011 ▸) with permission from Nature Publishing Group.

The high sensitivity of scattering cross-sections to composition is also an advantage when counting the number of atoms in an atomic column with single-atom precision. To illustrate this, scattering cross-sections have been determined for the atomic columns of a nanosized Ag cluster embedded in an Al matrix (Van Aert *et al.*, 2011 ▸). Fig. 5 ▸(*a*) shows an aberration-corrected HAADF STEM image of such clusters viewed along the 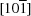 zone axis. Using the model-based approach explained above, the parameters of an empirical physics-based model have been estimated in the LS sense. For the cluster in the white boxed region, the refined model is shown in Fig. 5 ▸(*b*). Based on the estimated parameters, scattering cross-sections have been computed for each atomic column and these are shown in the histogram in Fig. 5 ▸(*d*). Since the thickness of the sample can be assumed to be constant over the particle area, substitution of an Al atom by an Ag atom leads to an increase in the estimated intensity. Owing to a combination of experimental detection noise and residual instabilities, broadened – rather than discrete – peaks are observed. Therefore, these results cannot be interpreted directly in terms of the number of atoms in a column. However, by evaluating the so-called integrated classification likelihood (ICL) criterion (McLachlan & Peel, 2000 ▸; De Backer *et al.*, 2013 ▸), as shown in Fig. 5 ▸(*e*), ten significant peaks are found and their positions are indicated by black dots in Fig. 5 ▸(*d*). From the estimated peak positions, the number of Ag atoms in each atomic column can be quantified, leading to the result shown in Fig. 5 ▸(*c*). This counting procedure has also been applied to the same Ag cluster viewed along the [100] direction, as shown in Figs. 5 ▸(*f*)–5 ▸(*h*). In §6 we will explain how atom-counting results obtained from different zone-axis orientations can be combined to retrieve the three-dimensional atomic structure. For example, the atom counts presented in Figs. 5 ▸(*c*) and 5 ▸(*h*) result in the reconstruction shown in Fig. 5 ▸(*i*).

**Figure 6:**
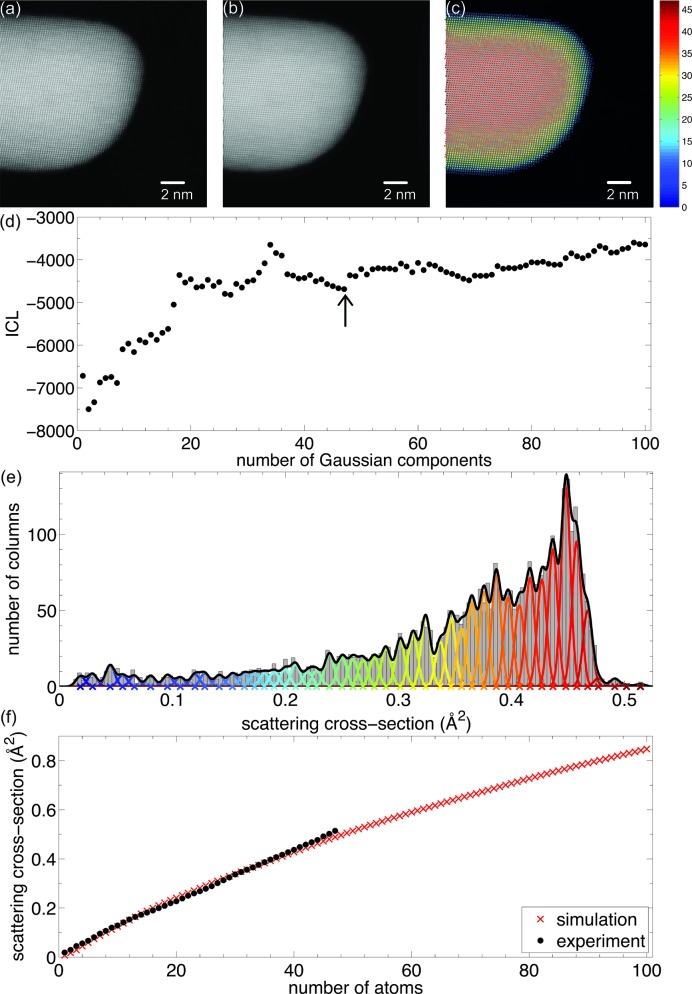
(*a*) Experimental HAADF STEM image of an Au nanorod. (*b*) Refined parameterized model. (*c*) The number of Au atoms per column. (*d*) ICL criterion evaluated as a function of the number of Gaussians in a mixture model. (*e*) Histogram of scattering cross-sections of the Au columns, together with the estimated mixture model and its individual components. (*f*) Comparison of experimental and simulated scattering cross-sections. Adapted with permission from Van Aert *et al.* (2013 ▸), copyright American Physical Society.

The most direct method for counting atoms is through comparison with image simulations (LeBeau *et al.*, 2010 ▸), but the main drawback with this approach is that systematic errors are difficult to detect. Indeed, the assignment of numbers of atoms will always find a match by comparing experimental scattering cross-section values or peak intensities with simulated values. The reliability then depends solely on the accuracy with which, for example, the detector inner and outer angles have been determined and the accuracy with which the simulations have been carried out. In comparison, the statistics-based method used to count the number of atoms shown in Fig. 5 ▸ is a simulation-free method. This approach is robust against systematic errors when two conditions are met: the number of experimental scattering cross-sections per unique thickness should be large enough and the spread of scattering cross-sections should be small enough compared with the difference between those of differing thicknesses (De Backer *et al.*, 2013 ▸). Ultimately, the simulations-based method and statistics-based method are combined into a hybrid approach. This allows one to compare both methods in an independent way and in this manner the accuracy of the obtained atom counts can be validated (Van Aert *et al.*, 2013 ▸; De Backer, Martinez *et al.*, 2015 ▸). An example analysis is presented in Fig. 6 ▸, showing the atom-counting analysis of an Au nanorod (Van Aert *et al.*, 2013 ▸). In this example, the intensities in the HAADF STEM image have been normalized with respect to the incident beam (Rosenauer *et al.*, 2009 ▸; Grieb *et al.*, 2012 ▸), allowing one to test the accuracy of the counting procedure. This validation step is required since more local minima are present in the ICL criterion shown in Fig. 6 ▸(*d*). Fig. 6 ▸(*f*) shows the experimental mean scattering cross-sections – corresponding to the component locations in Fig. 6 ▸(*e*) – together with the scattering cross-sections estimated from frozen phonon calculations using the *STEMsim* program under the same experimental conditions (Rosenauer & Schowalter, 2008 ▸). The excellent match of the experimental and simulated scattering cross-sections within the expected 5–10% error range validates the accuracy of the obtained atom counts (LeBeau *et al.*, 2010 ▸; Rosenauer *et al.*, 2011 ▸). The precision of the atom counts is limited by the unavoidable presence of noise in the experimental images, resulting in overlap of the Gaussian components as shown in Fig. 6 ▸(*e*). When the overlap increases, the probability of assigning an incorrect number of atoms will increase. In this example, the probability of having an error of one atom is only 20%, whereas the number of atoms of 80% of all columns can be determined without error. The combination of a simulation-based and a statistics-based method thus allows for reliable atom counting with single-atom sensitivity.

## 6. Atomic resolution in three dimensions   

As described in the previous sections of this feature article, new developments within the field of TEM enable the investigation of nanostructures at the atomic scale. Structural as well as chemical information can be extracted in a quantitative manner. However, such images are mostly two-dimensional projections of a three-dimensional object. To overcome this limitation, three-dimensional imaging by TEM or electron tomography can be used. Atomic resolution in three-dimensions has been the ultimate goal in the field of electron tomography during the past few years. The underlying theory for atomic-resolution tomography has been well understood (Saghi *et al.*, 2009 ▸; Jinschek *et al.*, 2008 ▸), but nevertheless it has been challenging to obtain the first experimental results. A first approach is based on the acquisition of a limited number of HAADF STEM images that are acquired along different zone axes (Van Aert *et al.*, 2011 ▸). As illustrated in §5, advanced quantification methods enable one to count the number of atoms in an atomic column from a two-dimensional (HA)ADF STEM image. In a next step, such atom-counting results can be used as input for discrete tomography. The discreteness that is exploited here is the fact that crystals can be thought of as discrete assemblies of atoms (Jinschek *et al.*, 2008 ▸). In this manner, a very limited number of two-dimensional images is sufficient to obtain a three-dimensional reconstruction with atomic resolution. This approach was applied to Ag clusters embedded in an Al matrix, as illustrated in Fig. 5 ▸(*i*) (Van Aert *et al.*, 2011 ▸). A three-dimensional reconstruction was obtained using only two HAADF STEM images. An excellent match was found when comparing the three-dimensional reconstruction with additional projection images that were acquired along different zone axes. In a similar manner, the core of a free-standing PbSe–CdSe core–shell nanorod could be reconstructed in three-dimensions (Bals *et al.*, 2011 ▸).

**Figure 7:**
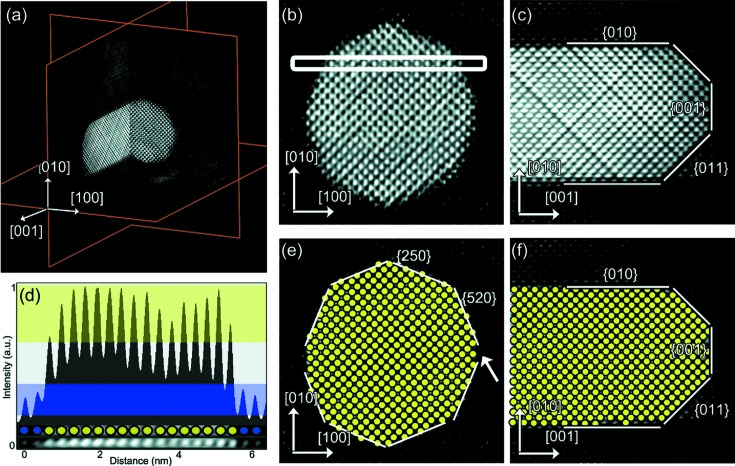
(*a*) Three orthogonal slices through the three-dimensional reconstruction show the core–shell structure of an Au@Ag nanorod. (*b*)–(*c*) Detailed views of slices through the reconstruction perpendicular and parallel to the major axis of the nanorod. (*d*) Intensity profile along the direction indicated by the white rectangle in part (*b*). (*e*)–(*f*) Slices corresponding to parts (*b*) and (*c*), in which each Au atom is indicated by a yellow disc. Interfacial planes are indicated. Reproduced from Goris *et al.* (2013 ▸) with permission from the American Chemical Society.

The discrete approach that was used in these studies assumes that the atoms are situated on a (fixed) face-centred cubic lattice and that the particle contains no holes. These assumptions provide a decent start for the investigations described above, but deviations from a fixed grid, caused by defects, strain or lattice relaxation, are exactly the parameters that determine the physical properties of nanomaterials. Compressive sensing-based tomography has shown its ability to provide an adequate solution for problems that can be represented in the form of a sparse representation (Donoho, 2006*a* ▸,*b* ▸; Saghi *et al.*, 2011 ▸; Goris, Bals *et al.*, 2012 ▸; Goris, Van den Broek *et al.*, 2012 ▸; Leary *et al.*, 2013 ▸; Thomas *et al.*, 2013 ▸). At the atomic scale, the approach exploits the sparsity of the object, since most of the voxels that need to be reconstructed correspond to vacuum and only a limited number of voxels are occupied by atoms (Goris, Bals *et al.*, 2012 ▸). An important advantage of this approach is that the actual positions of the atoms can be revealed without using assumptions concerning the crystal structure. This approach has been applied to reconstruct the structure of Au nanorods (Goris, Bals *et al.*, 2012 ▸) and to reconstruct the atom type of individual atoms in Au@Ag nanoparticles, as shown in Fig. 7 ▸ (Goris *et al.*, 2013 ▸). Such bimetallic particles often provide novel properties compared with their monometallic counterparts (Henglein, 2000 ▸; Hodak *et al.*, 2000 ▸; Tedsree *et al.*, 2011 ▸; Cortie & McDonagh, 2011 ▸). To understand these properties, a complete three-dimensional characterization is often required where the exact positioning of the different chemical elements is crucial, especially at the interfaces. Owing to the atomic number *Z*-dependence of HAADF STEM intensities, the position and atom type of each atom have been determined from five high-resolution images acquired along different major zone axes. Using statistical parameter estimation theory, the parameters of an incoherent imaging model have been estimated and the resulting models used as input for the compressive sensing-based algorithm. A detailed analysis of the position and atom type in a core–shell bimetallic nanorod was performed using orthogonal slices through the three-dimensional reconstruction, as shown in Fig. 7 ▸ (Goris *et al.*, 2013 ▸). Individual Ag and Au atoms can be distinguished, even at the metal/metal interface, by comparing their relative intensities. An intensity profile was acquired along the direction indicated by the white rectangular box in Fig. 7 ▸(*b*) and shown in Fig. 7 ▸(*d*), from which it is clear that Au and Ag atoms can indeed be identified from their intensities using a threshold value. In this manner, each atom in the cross-sections shown in Figs. 7 ▸(*b*) and 7 ▸(*c*) was assigned to be either Ag or Au. The results are shown in Figs. 7 ▸(*e*) and 7 ▸(*f*) and lead to correct indexing of the type of interfacial plane.

**Figure 8:**
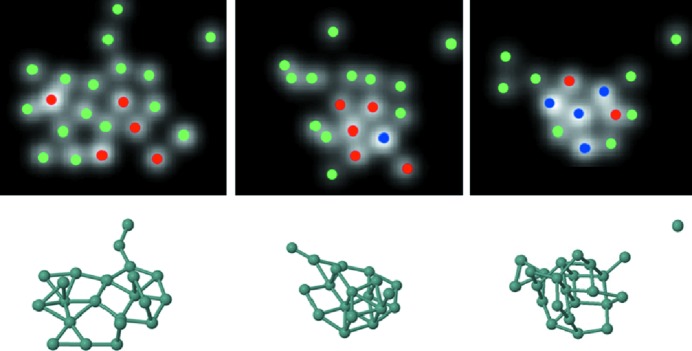
The top row presents the statistical counting results for three different configurations of an ultra-small Ge cluster. Green, red and blue dots correspond to one, two and three atoms, respectively. The results of the *ab initio* calculations are shown at the bottom. Reproduced from Bals *et al.* (2012 ▸) with permission from Nature Publishing Group.

Ultra-small nanoparticles or clusters, having sizes below 1 nm, form a challenging subject of investigation. In particular, the characterization of their structure is far from straightforward. At the same time, however, there is a clear need for a complete characterization in three-dimensions since these materials can no longer be considered as periodic objects. One of the main bottlenecks is that these clusters may rotate or show structural changes during investigation by TEM (Li *et al.*, 2007 ▸). Obviously, conventional electron tomography methods, even those that are based on a limited number of projections, can no longer be applied. On the other hand, the intrinsic energy transfer from the electron beam to the cluster can be considered as a unique possibility to investigate the transformation between energetically excited configurations of the same cluster. This idea was exploited to study the dynamic behaviour of ultra-small Ge clusters consisting of less than 25 atoms (Bals *et al.*, 2012 ▸). Two-dimensional image series were collected using aberration-corrected HAADF STEM and selected frames were analysed using statistical parameter estimation theory. In this manner, the number of atoms at each position could be determined, as illustrated in Fig. 8 ▸. In order to extract three-dimensional structural information from these images without using prior knowledge of the structure, *ab initio* calculations were carried out. Several starting configurations were constructed that are all in agreement with the experimental two-dimensional projection images. Although all of the cluster configurations stay relatively close to their starting structure after full relaxation, only those configurations in which a planar base structure was assumed were found to be still compatible with the two-dimensional experimental images. In this manner, reliable three-dimensional structural models are obtained for these small clusters and the transformation of a predominantly two-dimensional configuration into a compact three-dimensional configuration can also be characterized.

## 7. Conclusions and outlook   

The use of statistical parameter estimation techniques in the field of electron microscopy is becoming increasingly important since it allows one to determine unknown structures quantitatively on a local scale. The theory of parameter estimation is well established and applications are becoming routine, partly through improvements in the underlying algorithms to estimate unknown structure parameters, and partly through the increase in computational power that allows fast processing and analysis. Applications in the field of high-resolution (S)TEM show how statistical parameter estimation techniques can be used to overcome the traditional limits set by modern electron microscopy. The precision that can be achieved in this quantitative manner far exceeds the resolution performance of the instrument. The characterization limits are therefore no longer imposed by the quality of the lenses but are determined by the underlying physical principles. Structural, chemical, electronic and magnetic information can be obtained at the atomic scale. As demonstrated in this feature article, not only can quantitative structure determination be carried out in two-dimensions, but also three-dimensional analyses are currently becoming standard.

The examples discussed in this feature article demonstrate that statistical parameter estimation methods have been applied successfully to nanostructures which are relatively stable under the incoming electron beam, and therefore the atomic structure under investigation can be assumed to remain unchanged under illumination with high electron doses. However, radiation damage becomes increasingly relevant not only in biological studies but also in the study of nanostructures (Meyer *et al.*, 2014 ▸). An important challenge that remains is therefore to push the development of quantitative methods toward its fundamental limits. Ultimately, the goal is to measure the atom positions of beam-sensitive nanostructures with picometre precision and to discern between adjacent atom types. This is very challenging and to reach this goal the allowable electron dose needs to be used in the most optimal way. Indeed, every incoming electron counts and therefore needs to carry as much quantitative structural information as possible. For that purpose, the microscope and detector settings will be optimized using the principles of statistical experimental design (den Dekker *et al.*, 1999 ▸, 2001 ▸, 2013 ▸; Van Aert, den Dekker, Van Dyck & van den Bos, 2002 ▸; Van Aert, den Dekker & Van Dyck, 2004 ▸; Van Aert, den Dekker *et al.*, 2004 ▸). This becomes increasingly important in an era where new data collection geometries are emerging (Shibata *et al.*, 2010 ▸; Yang *et al.*, 2015 ▸). Therefore, the use of expressions representing the attainable precision, as discussed in this feature article, can be used to optimize the experimental design. Statistical experimental design can be defined as the selection of free variables in an experiment to improve the precision of the measured parameters. By calculating the attainable precision, the experimenter is able to verify whether, for a given experimental design, the precision is sufficient for the purpose at hand. If not, the experiment design has to be optimized so as to attain maximum precision. This will allow a significant reduction in the incoming electron dose to achieve maximum attainable precision or detectability. Finally, when lowering the incoming electron dose, it is expected that the use of a complete maximum likelihood method to estimate unknown structure parameters will be of great help (den Dekker *et al.*, 2005 ▸), in which the image-formation process is described using first-principles quantum mechanical models instead of simplified empirical models. Furthermore, an accurate description of the detector and noise properties, taking correlations between neighbouring pixel values into account, would then be required (Niermann *et al.*, 2012 ▸; Lubk *et al.*, 2012 ▸). The use of a complete maximum likelihood estimation method will be computationally demanding, in which case graphical processing unit (GPU) computing strategies to reduce the total computing time will be very welcome (Dwyer, 2010 ▸; Lobato & Van Dyck, 2015 ▸). However, if successful, we will be able to measure unknown structure parameters as accurately and as precisely as possible using a given electron dose.

In conclusion, the possibilities of statistical parameter estimation theory in the field of electron microscopy have been shown to be successful in the study of many materials problems so far. Recent developments to explore the capabilities of new detector geometries will certainly open up a whole new range of possibilities to understand and characterize beam-sensitive nanostructures in particular.
